# Comparison of the effects of open vs. closed skill exercise on the acute and chronic BDNF, IGF-1 and IL-6 response in older healthy adults

**DOI:** 10.1186/s12868-021-00675-8

**Published:** 2021-11-25

**Authors:** Tom Behrendt, Franziska Kirschnick, Lasse Kröger, Phillip Beileke, Maxim Rezepin, Tanja Brigadski, Volkmar Leßmann, Lutz Schega

**Affiliations:** 1 Chair for Health and Physical Activity, Department of Sport Science, Faculty of Humanities, Otto-von-Guericke University Magdeburg, Magdeburg, Germany; 2 Department of Internal Medicine, Division of Cardiology and Angiology, University Hospital Magdeburg, Magdeburg, Germany; 3 Department of Informatics and Microsystem Technology, University of Applied Sciences, Kaiserslautern, Germany; 4 Institute of Physiology, Medical Faculty, Otto-von-Guericke University Magdeburg, Magdeburg, Germany; 5 Center for Behavioral Brain Sciences, Magdeburg, Germany

**Keywords:** Open skill exercise, Closed skill exercise, Brain-derived neurotrophic factor (BDNF), Insulin-like growth factor 1 (IGF-1), Interleukin-6 (IL-6), Aging

## Abstract

**Background:**

Accumulating evidence shows that physical exercise has a positive effect on the release of neurotrophic factors and myokines. However, evidence regarding the optimal type of physical exercise for these release is still lacking. The aim of this study was to assess the acute and chronic effects of open-skill exercise (OSE) compared to closed-skill exercise (CSE) on serum and plasma levels of brain derived neurotrophic factor (BDNF_S_, BDNF_P_), and serum levels of insulin like growth factor 1 (IGF-1), and interleukin 6 (IL-6) in healthy older adults.

**Methods:**

To investigate acute effects, thirty-eight participants were randomly assigned to either an intervention (badminton (aOSE) and bicycling (aCSE), n  = 24, 65.83 ± 5.98 years) or control group (reading (CG), n  = 14, 67.07 ± 2.37 years). Blood samples were taken immediately before and 5 min after each condition. During each condition, heart rate was monitored. The mean heart rate of aOSE and aCSE were equivalent (65 ± 5% of heart rate reserve). In a subsequent 12-week training-intervention, twenty-two participants were randomly assigned to either a sport-games (cOSE, n  = 6, 64.50 ± 6.32) or a strength-endurance training (cCSE, n  = 9, 64.89 ± 3.51) group to assess for chronic effects. Training intensity for both groups was adjusted to a subjective perceived exertion using the CR-10 scale (value 7). Blood samples were taken within one day after the training-intervention.

**Results:**

BDNF_S_, BDNF_P_, IGF-1, and IL-6 levels increased after a single exercise session of 30 min. After 12 weeks of training BDNF_S_ and IL-6 levels were elevated, whereas IGF-1 levels were reduced in both groups. However, only in the cOSE group these changes were significant. We could not find any significant differences between the exercise types.

**Conclusion:**

Our results indicate that both exercise types are efficient to acutely increase BDNF_S_, BDNF_P_, IGF-1 and IL-6 serum levels in healthy older adults. Additionally, our results tend to support that OSE is more effective for improving basal BDNF_S_ levels after 12 weeks of training.

## Background

Aging is associated with changes in brain structure and function [1–4] and in turn, with an increased risk of developing cognitive impairments and neurodegenerative diseases (e.g.,, dementia) [5–7]. In this context, it has been shown that physical exercise (defined as a specific, planned and structured form of physical activity, which leads to acute (transient) effects [8–10]) and/or physical training (defined as repeated bouts of physical exercise, which leads to chronic (long-term) effects [8, 10]) could be an appropriate approach to maintain or enhance physiological brain functions, thereby helping to prevent neurodegenerative diseases in the elderly population [11–19].

Although the exact underlying neurobiological mechanisms are not yet fully understood, it is assumed that the beneficial effects of physical exercise and/or physical training on cognition are based on effects that can be observed at multiple levels (i.e., molecular and cellular changes, structural and functional changes, socioemotional changes) [20–22]. At the molecular level, different neurotrophic factors and myokines like brain-derived neurotrophic factor (BDNF), insulin-like growth factor 1 (IGF-1), and interleukin-6 (IL-6) are currently described as possible key factors that are up-regulated and released in response to acute physical exercise [23–29]. According to the “neurotrophic hypotheses” [30, 31], in long-term the exercise-related release of neurotrophic factors can promote functional and structural brain changes, that in turn might contribute to the maintenance or increase of cognitive functions [32, 33]. In this regard, it has been shown that BDNF promotes various processes of neuroplasticity, such as neurogenesis, neuronal differentiation, synaptogenesis and neuronal protection [34–36]. Furthermore, BDNF is crucially involved in processes of synaptic plasticity like long-term potentiation throughout the brain and thus regulates memory formation and learning processes [37–41]. Along these lines, higher serum levels of BDNF are positively associated with increased hippocampal volume in humans [42]. There is also evidence that an increase in IGF-1 improves neurogenesis [43–45], the maintenance and reshaping of brain vessels (angiogenesis), as well as the survival of neurons [46]. With regard to neurocognitive changes, IL-6 is involved in the homeostatic control of memory mechanisms and neurophysiological processes in the brain [47] and stimulates the production of anti-inflammatory cytokines such as IL-1ra and IL-10 [48]. However, current evidence suggests that the expression of neuroproteins and/or myokines (e.g.,, BDNF, IGF-1, IL-6) depends on exercise variables (e.g.,, exercise intensity, exercise duration, type of physical exercise) and/or training variables (e.g.,, frequency, density, duration) and training principles (e.g.,, progression, specificity, reversibility) [16, 49–52].

With regard to the type of physical exercises, there exist various classification approaches in the literature. Exercise types can be classified based on the predictability of the performing environment and the exercise complexity (motor and/or cognitive demands) [53, 54]. According to this differentiation, exercise types can be classified into (i) open skill exercise (OSE) and (ii) closed skill exercise (CSE). OSE (e.g., badminton, table tennis) are performed in dynamic, externally-paced, and more unpredictable environments, while CSE (e.g., running, bicycling) includes relatively consistent, self-adjustable and more predictable environments [50]. A recent review by Gu et al. [50] that compared the effects of OSE versus CSE on cognitive functions, showed that OSE led to greater improvements in cognitive functions in both children and older adults. However, the majority of the studies in this review were observational studies. So it is not clear whether OSE is more beneficial to brain health than CSE or whether people with better status of cognitive functions prefer this type of exercise. These results are confirmed by the meta-analysis of Zhu et al. [55]. The authors show that, compared with CSE, OSE is more advantageous in improving cognitive functions, especially with respect to executive functions such as inhibition and cognitive flexibility. However, only 4 out of 19 studies included in this meta-analysis were intervention studies. Excluding the cross-sectional studies, no significant differences could be found between the two exercise types [55]. In this context, Hung et al. [56] investigated the acute effect of OSE and CSE on the BDNF concentration in the blood of young males. The results of this study showed that OSE (badminton) leads to a stronger BDNF release than CSE (running). However, the observed acute effects found in the study of Hung et al. [56] cannot be transferred to chronic effects or other cohorts, e.g., older people.

To further investigate the acute and chronic effects of OSE versus CSE on the release of neurotrophic proteins and myokines, we compared the exercise and training related changes in blood levels of BDNF, IGF-1 and IL-6 in response to the two different types of exercise in healthy older adults.

## Methods

### Study design

**Fig. 1:**
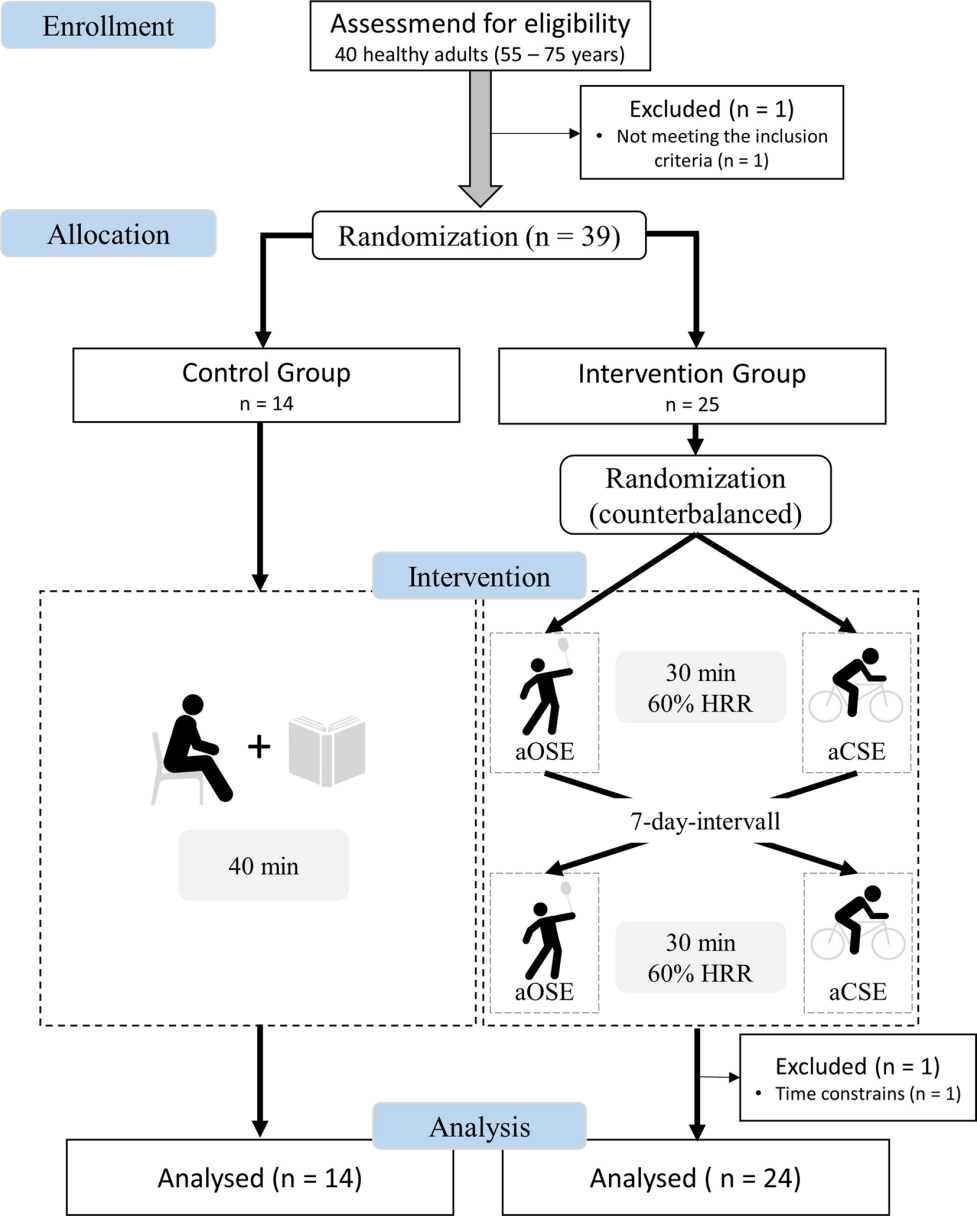
Study design for the first investigation (acute effects)

**Fig. 2:**
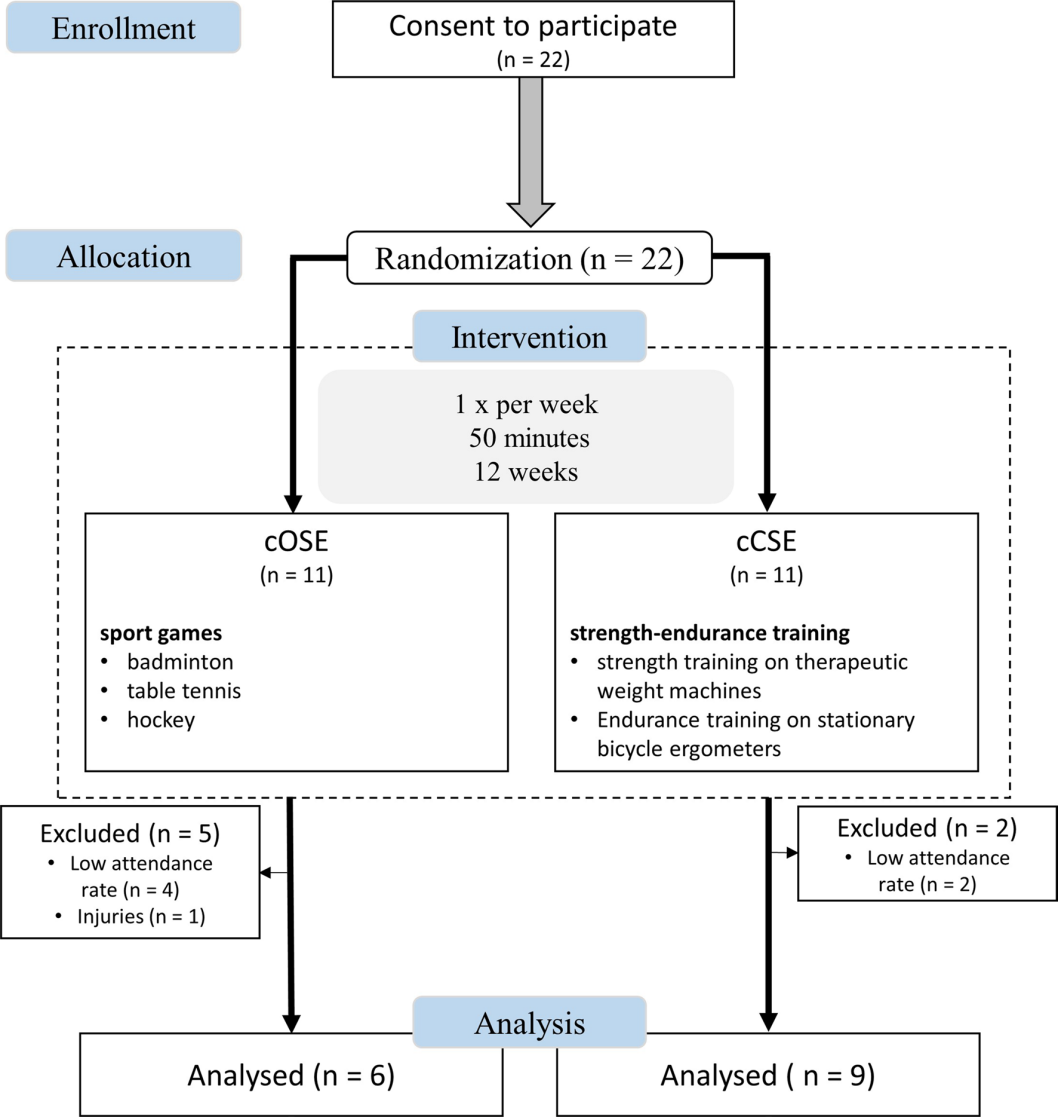
Study design for the second investigation (chronic effects)

The present study consisted of two investigations with different study designs (see Figs. 1, 2) to determine the (i) acute effects (after a single exercise session) and (ii) chronic effects (after a training period consisting of several acute exercise sessions) of two different exercise types (OSE vs. CSE) on the blood levels of BDNF (plasma and serum), IGF-1, and IL-6. To assess acute exercise-related effects, we used a 3 group  × 2 time points (pre and post exercise) cross-over design. Following the first investigation, a 12 week-long training intervention study with a 2 group  × 2 time (pre and post training intervention) parallel group design was conducted between April and June 2019 to determine the chronic effects. The group allocation in both groups was randomized. Due to the nature of physical exercise interventions, it was not possible to blind the participants and the trainers. Outcome assessors including the medical doctor who took the blood samples and the laboratorian who analysed the blood samples for BDNF, IGF-1 and IL-6 concentration were blinded because they had no knowledge of the subjects’ group allocation. The study is in accordance with the principles stated in the declaration of Helsinki and was reviewed and approved by the board of the ethical committee of the Otto-von-Guericke University Magdeburg (29/19).

To investigate acute effects three separate appointments were carried out. At the first appointment, the participants were informed about the aim, purpose and procedure of the study and gave their written informed consent for voluntary participation. After consent was obtained, body height and body mass were measured using a standard stadiometer (Seca, Switzerland). Thereafter, the participants’ physical activity and mental status were determined using the German questionnaire “Freiburger Fragebogen zur körperlichen Aktivität” (FFkA) [57] and the Mini-Mental State Examination (MMSE) [58], respectively. The FFkA determined the health related activity including basic activity, extracurricular activity and activity in sports retrospective for 1 week (in hours per week). Following this, participants lay down in a quiet room for 15 min while the heart rate was measured using a heart rate monitor (RS800cx, Polar, Finland). The resting heart rate was defined as the mean value of the last 5 min. Participants were then randomly assigned in either an intervention group or a control group. After screening, participants from the intervention group individually attended to the laboratory for two exercise sessions (OSE and CSE session) with a 7-day interval between sessions. The order in which the participants either performed the OSE or the CSE session was randomized and counterbalanced. The control group visited the laboratory for only one additional measurement appointment. Thus, the first investigation (assessing the acute effects) consists of three groups: (i) acute OSE (aOSE), (ii) acute CSE (aCSE) and (iii) control group (CG). After the first investigation had been completed, we conducted a 12-week training intervention to compare the chronic effects of OSE versus CSE. There were at least 8 days between the last acute exercise session of the first investigation and the first training session of the second investigation. Therefore, we designed two active groups: (i) chronic OSE (cOSE) and (ii) chronic CSE (cCSE) without an inactive control group.

### Participants

Forty healthy elderly volunteers aged 55–75 years were recruited by a newsletter announcement and screened for their eligibility to participate. Exclusion criteria were defined as follows: participating in any regular exercise program, cognitive impairments as detected by the MMSE, any history of several cardiovascular or metabolic diseases, and reduced and uncorrected vision. Thirty-nine participants met the inclusion criteria and were included in our study.

Regarding the first investigation assessing the acute effects of a single exercise session, 25 of the remaining participants were assigned to the intervention group and 14 participants were assigned to the control group. One participant from the intervention group left the study due to time constrains. Finally, data sets of 38 participants (24 from the intervention group and 14 from the control group) were analysed (see Fig. 1). The participants’ characteristics of both groups are presented in Table 1.

**Table 1:** Characteristics on the participants of the active intervention group (aOSE and aCSE) and the inactive control group (CG) at baseline

Group N (female/male)	Intervention Group 24 (12/12)	CG 14 (6/8)
Measure	M (SD)	M (SD)
Age (years)	65.83 (5.98)	67.07 (2.37)
< 65 years [N (%)]	9 (37.5%)	2 (14.3%)
≥ 65 years [N (%)]	15 (62.5%)	12 (85.7%)
Weight (kg)	84.05 (16.51)	76.84 (12.69)
Height (m)	1.73 (0.10)	1.68 (0.08)
BMI (kg/m^2^)	28.08 (4.37)	27.24 (4.41)
kcal/week	2047.23 (1674.41)	2313.03 (2118.45)
Resting HR (bpm)	68.25 (9.50)	70.00 (9.00)
Educational achievement (N)		
Secondary education	7 (29.2%)	6 (42.9%)
High school	5 (20.8%)	2 (14.3%)
University	10 (41.7%)	3 (21.4%)
Academic degree	2 (8.3%)	3 (21. 4%)
MMSE	28.8 (1.2)	29.1 (0.9)

*BMI* body mass index; *HR* heart rate

Following the first investigation, 22 participants from the intervention group and the control group voluntarily agreed to participate in the long-term intervention study to assess chronical effects. Participants were randomly allocated to either the cCSE group or the cOSE group. After 12 weeks of training, we were able to evaluate the data of 15 participants (6 from the cOSE group and 9 from the cCSE group). Seven participants had to be excluded due to injuries (cOSE group: n  = 1) and insufficient attendance rates (cCSE group: n  = 4, cOSE group: n  = 2). The anthropometric characteristics (age, weight, height, and body mass index) of the remaining participants are depicted in Table 2.

**Table 2:** Characteristics on the participants of the cOSE group and the cCSE group at baseline

Group N (female/male)	cOSE 6 (4/2)	cCSE 9 (6/3)
Measure	M (SD)	M (SD)
Age (years)	64.50 (6.32)	64.89 (3.51)
< 65 years [N (%)]	2 (33.3%)	4 (44.4%)
≥ 65 years [N (%)]	4 (66.7%)	5 (55. %)
Weight (kg)	76.05 (11.52)	86.02 (28.68)*
Height (m)	1.66 (0.05)	1.73 (0.05)
BMI (kg/m^2^)	27.58 (4.48)	28.68 (5.28)
kcal/week	2407.28 (1973.127)	1073.10 (670.21)
Resting HR (bpm)	67.67 (5.57)	67.56 (10.48)
Educational achievement (N)		
Secondary education	3 (50.0%)	2 (22.2%)
High school	1 (16.7%)	1 (11.1%)
University	2 (33.3%)	4 (44.4%)
Academic degree	0 (0.0%)	2 (22. 2%)
MMSE	28.7 (1.8)	29.0 (0.9)

*BMI* body mass index

Asterisk indicate significant difference between groups (*0.050 >  p  ≥ 0.001)

### Procedure

#### First investigation: acute effects

The procedure for the first investigation is depicted in Fig. 1. Participants from the intervention group were instructed to perform either a single/acute OSE session (aOSE group, badminton) or a single/acute CSE session (aCSE group, bicycling) (i.e., participants who started with the OSE session as the first intervention performed the CSE session during the second measurement appointment and vice versa). Both exercise sessions consisted of an identical 5-min warm up, followed by 30 min of the main exercise (badminton or bicycling) and an identical 5-min cool down. During the exercise sessions, heart rate was continuously recorded and controlled using a heart rate monitor (RS800cx, Polar, Finland). In the aOSE group (badminton session), the participants played individually against one of the two experienced instructors (i.e., 1 versus 1) without any breaks (e.g., for changeover). Scores were not recorded and no match-winner was determined. The single session of the aCSE group (bicycling session) was performed on a stationary bicycle ergometer (Kardiomed, Proxomed^®^, Germany) at a cadence of 70–80 revolutions per minute. The average session intensity was set to 60 ± 5% of each participant’s individual heart rate reserve (HRR). The HRR was defined as the difference between the maximum heart rate (HR_max_) and the resting heart rate (HR_rest_) (HRR = HR_max_ − HR_rest_). The HR_max_ is calculated using the formula $${HR}_{max}=205.8-0.685\times age$$ [59]. The target heart rate for the exercise sessions was calculated using the Karvonen formula $$target heart rate=\left(HRR\times 60\%\right)+{HR}_{rest}$$ [60]. Participants from the CG rested in a sitting position for 40 min in a quiet room and were asked to read in a magazine with sport science topics.

Blood samples were obtained at two time points: immediately before (T1) and 5 min after (T2) the respective test condition (badminton, bicycling or reading). Furthermore, ratings of perceived exertion (RPE) measures were obtained at rest and immediately after completing the respective test condition using the RPE-scale, which ranges from 6 to 20 with 6 for minimal effort and 20 for maximal effort [61]. In addition, for both, the aOSE and aCSE group, an end-to-end capillary of 10 μl of capillary blood was taken from the right hyperemic earlobe before and after exercising to determine the blood lactate concentration.

#### Second investigation: chronic effects

The procedure for the second investigation is depicted in Fig. 2. The training sessions of the cCSE group as well as the cOSE group took place once a week, lasting 50 min per session (5 min warm up, followed by 40 min of the main exercise and 5 min cool down), and were provided for 12 weeks. All training sessions were conducted by a sports coach who demonstrated the exercises.

The cOSE group practiced different sport games such as badminton, hockey and table tennis with a focus on learning specific techniques and variants of the respective sport game. In each individual session the participants either learned a new technique or were confronted with a new variation of the game.

The main part of the training sessions of the cCSE group consisted of two exercise blocks: (i) strength training and (ii) endurance training. Each exercise block lasted 20 min. The strength training involved exercises comprised alternating movements (e.g., leg-press, leg-extension, leg-flexion, pull-down, rowing, chest-press, triceps-press, butterfly, upper body-flexion, upper body-extension, upper body-rotation) and was performed at therapeutic weight machines (compass 530, Proxomed^®^, Germany) with predicted motion range and direction to keep the coordinative requirements as low as possible. The intensity of the resistance training was determined by the following variables: number of exercises per unit: 4; sets per exercise: 3; repetitions per set: 15; repetition velocity: 2–0–2–0 (2 s concentric, 0 s isometric, 2 s eccentric, 0 s isometric; time under tension: 60 s); inter-set rest period: 30 s; inter-exercise rest period: 2 min. The endurance training was performed on stationary bicycle ergometers with a cadence between 70 and 80 revolutions per minute. Compared to our first investigation (acute effects), we changed our approach to define exercise intensity because of methodological reasons (e.g., number of people exercising at the same time and exercise mode). For the second investigation (chronic effects), the intensity of the training was controlled and adjusted by an inductive approach using the Category Ratio-10 Scale (CR-10 Scale) [62]. Participants were asked to rate their perceived exertion following each exercise (including the endurance training). The target intensity was defined as “very strong” (7 on the CR-10 Scale). If the participants indicated values above or below the target intensity, the training load was adjusted by the trainer for the next training session. The participants were not informed about the target intensity so that they would not be influenced in their subjective perception.

Blood samples were taken within one day after (T3) the intervention to quantify basal changes (from T1 to T3).

### Blood sampling

Venous blood samples were taken by a medical doctor at the above mentioned time points (T1, T2 and T3) from the median cubital vein or the cephalic vein. The procedure of the blood collection was identical for all participants and was performed by the same persons. The blood samples were collected in two vacutainers with separating gel and coagulation activator (BD Vacutainer^®^ SST™ II Advance, green, 8.5 ml) and one vacutainer with li-heparin (BD Vacutainer^®^ LiHeparin, yellow, 4.0 ml). The vacutainer with li-heparin was used to determine the plasma level of BDNF. The vacutainers with separating gel and coagulation activator were used to analyze the serum levels of BDNF, IGF-1 and IL-6. Immediately after blood collection, blood samples were swirled head down for ten times. Thereafter, the serum samples were rested for 30 min at room temperature whereas the plasma sample rested for 10 min on ice. All blood samples were centrifuged at 2000*g* for 15 min. 300 µl of the supernatant fluid were then extracted and stored at − 80 °C. To analyzed the serum and plasma concentration of BDNF we used the BDNF DuoSet ELISA kit (R&D Systems^®^, Wiesbaden, Germany). Serum IL-6 and IGF-2 levels were quantified using a chemiluminescent immunometric assay (for IL6: IMMULITE^®^ 2000; Siemens Medical Solution Diagnostics, for IGF-1: IDS-iSYS; Immunodiagnostic Systems). The samples were processed accordingly to the kit instructions.

### Statistical analysis

Data were tested for normal distribution and homogeneity of variance using the Kolmogorov–Smirnov-Test and Levene’s test, respectively. For normally distributed data, a 2 (time: pre-test, post-test)  × 3 (group: aOSE, aCSE, CG) repeated-measures ANOVA was used to check for interaction effects and main effects (main group effect and main time effect). To identify differences between groups during a measurement time point (group effect), we performed post hoc t test. If significant main time effects were detected, a one-way ANOVA with repeated measures was performed to identify time effects within the groups. The post-hoc tests were adjusted by Bonferroni correction [63]. To clarify the practical relevance of the results, the effect size partial eta squared (η_p_^2^) were reported [64]. Group differences in age, weight, height, BMI, physical activity level, and resting HR were analyzed using an independent t test. For not normally distributed data, differences between measurement time points within a group (time effect) and differences between groups within a measurement time point (group effect) were analyzed with a Wilcoxon-test and Mann–Whitney *U* test, respectively. For statistically significant results, the effect size for these non-parametric tests were calculated using the formula: $r= \left|z\right|/\sqrt{n}$ with r  ≥ 0.5 rates a large effect, 0.5 <  r  ≥ 0.3 rates a medium effect and 0.3 <  r  ≥ 0.1 rates a small effect. All statistical analyses were calculated with SPSS Statistics 25 (IBM, Inc., Chicago). The level of significance was set to α  = 0.050.

## Results

### Acute effects

**Fig. 3:**
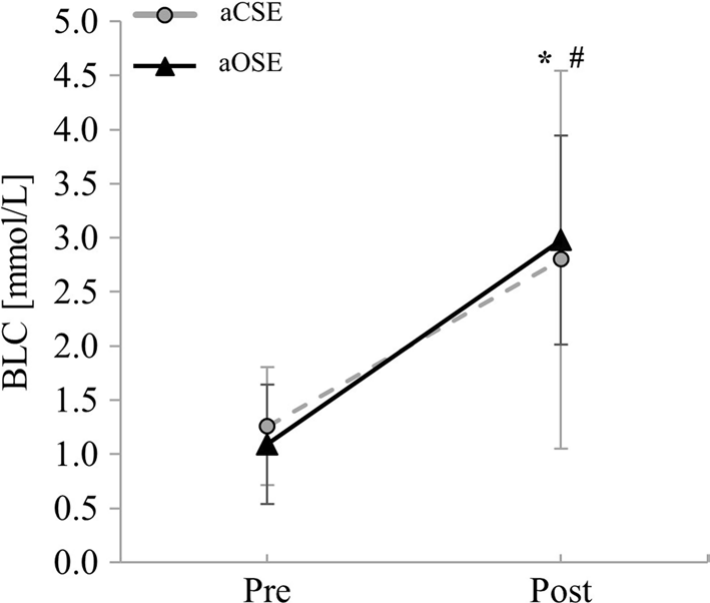
Means and standard deviations of the blood lactate concentrations for the acute open-skill exercise group (aOSE: badminton) and the acute closed-skill exercise group (aCSE: bicycling) at pre-test and post-test. Asterisk or number sign indicates a significant difference between pre-test and post-test for aOSE (*p < 0.050) and aCSE (^#^p < 0.050), respectively

At baseline, no differences in age, weight, height, body mass index, activity level, and resting heart rate were observed between the active intervention group (aOSE and aCSE) and the inactive control group. Results also showed no differences for the average exercise heart rate between aOSE (129.21 ± 12.96 bpm) and aCSE (124.50 ± 7.15 bpm). For RPE, no differences between groups were found at pre-test (aOSE: 9.0 ± 3.5; aCSE: 9.0 ± 2.3; CG: 9.0 ± 2.0). Significant time effects were observed for the aOSE (Z = 4.212, p < 0.001; 14.0 ± 2.0) and aCSE group (Z  = 4.217, p  < 0.001; 13.0 ± 2.0), indicating that participants had higher RPE values after exercising. In the post-test, significantly higher RPE values were found for the aOSE (Z  =  − 4.150, p  < 0.001) and aCSE group (Z  =  − 4.076, p  < 0.001) in comparison to the inactive CG (10.5 ± 2.0). Between aOSE and aCSE, however, no significant differences were found (Z = -0.414, p = 0.679). In addition, there was no significant interaction effect (F_1,23_  = 1.274, p  = 0.271, η_p_^2^  = 0.052) or group effect (F_1,23_  = 0.003, p  = 0.956, η_p_^2^  = 0.000) in blood lactate concentration. The time effect was significant (F_1,23_  = 45.695, p  < 0.001, η_p_^2^  = 0.665), with higher blood lactate concentrations at post-test (see Fig. 3).

#### Brain-derived neurotrophic factor

**Fig. 4:**
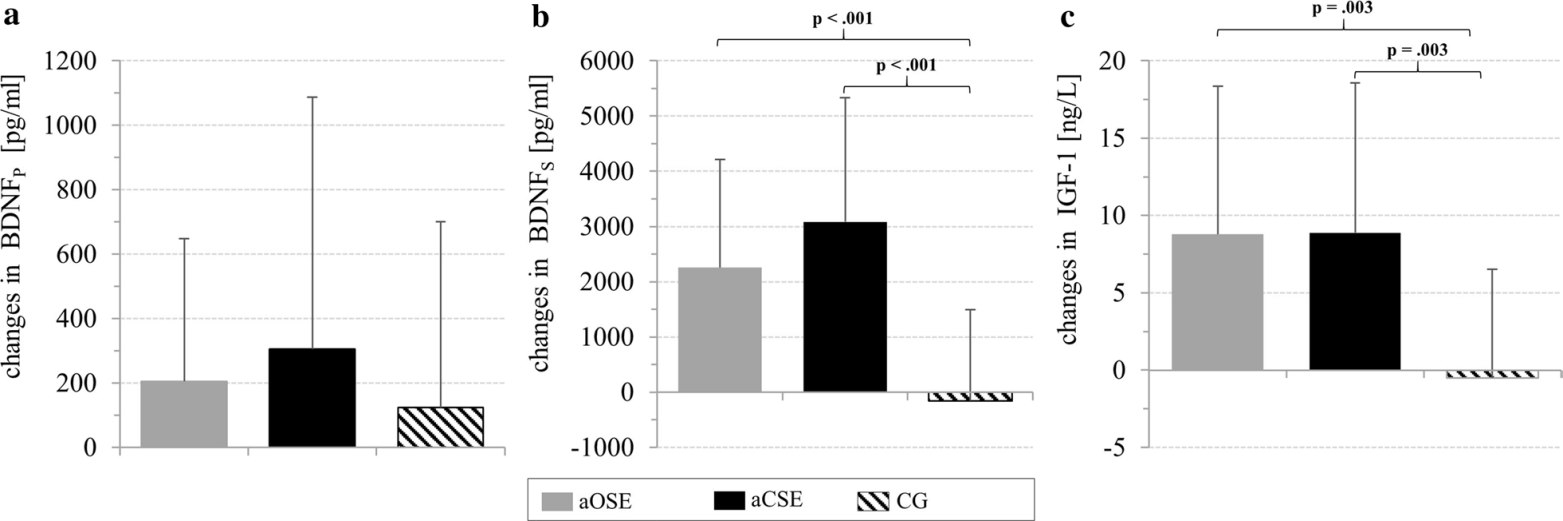
Means and standard deviations of absolute changes (pre-test–post-test) in **a** BDNF_P_, **b** BDNF_S_ and **c** serum IGF-1 for the acute open-skill exercise group (aOSE: badminton), the acute closed-skill exercise group (aCSE: bicycling) and the control group (CG: reading)

Outcome values of BDNF_P_ and BDNF_S_ are shown in Table 3. No interaction effect (F_2,58_  = 0.392, p  = 0.678, η_p_^2^  = 0.013) or main group effect (F_2,58_  = 1.039, p  = 0.360, η_p_^2^  = 0.035) could be observed for BDNF_P_. Nevertheless, we found a main time effect (F_1,58_  = 6.730, p  = 0.012, η_p_^2^  = 0.104) with significantly increased BDNF_P_ concentrations after the aOSE session (F_1,23_  = 5.135, p  = 0.033, η_p_^2^  = 0.183). No significant time effect, but a tendency, was observed for the aCSE session (F_1,23_  = 3.572, p  = 0.074, η_p_^2^  = 0.138). The absolute changes in BDNF_P_ showed no significant differences between groups (see Fig. 4). For BDNF_S,_ we found a significant interaction effect (F_2,58_  = 11.619, p  =  < 0.001, η_p_^2^  = 0.286) and main time effect (F_1,58_  = 42.567, p  < 0.001, η_p_^2^  = 0.423), while no main group effect occurred (F_2,58_  = 0.113, p  = 0.894, η_p_^2^  = 0.004). In both, the aOSE and the aCSE group, BDNF_S_ increased significantly from pre-test to post-test (F_1,23_  = 32.145, p  < 0.001, η_p_^2^  = 0.582 and F_1,23_  = 43.374, p  < 0.001, η_p_^2^  = 0.663, respectively). As shown in Fig. 4, the increase in BDNF_S_ was significantly higher in the aOSE and the aCSE group compared to the CG.

**Table 3:** Acute effects of OSE and CSE on blood levels of BDNF_P_, BDNF_S,_ IGF-1 and IL-6

Variable	Intervention group				CG	
	aOSE		aCSE			
	Pre	Post	Pre	Post	Pre	Post
BDNF_P_ (pg/ml)	1599.54 (738.57)	1804.63* (780.00)	1376.26 (621.73)	1682.04^#^ (807.15)	1825.43 (927.54)	1950.14 (1043.07)
BDNF_S_ (pg/ml)	27,525.00 (6781.45)	29,783.33** (7433.40)	26,473.91 (6317.31)	29,556.52** (6929.83)	27,685.71 (7380.52)	27,528.57 (6848.74)
IGF-1 (ng/ml)	183.33 (66.83)	192.13** (65.65)	192.42 (70.50)	201.29** (75.30)	174.50 (63.66)	174.00 (64.94)
IL-6 (ng/L)	2.15 (1.65)	3.25* (2.90)	1.60 (1.18)	2.75** (2.88)	1.55 (0.53)	1.60 (0.60)

Mean values (standard deviation) of BDNF_P_, BDNF_S_, and serum IGF-1—as well as median values (interquartile range) of serum IL-6 at pre-test and post-test

For BDNF_P_, BDNF_S_, and IGF-1, parametric tests were used. For IL-6, non-parametric tests were used

*BDNF* brain-derived neurotrophic factor; *IGF-1* insulin-like growth factor 1; *IL-6* interleukine-6

Asterisk indicate significant difference between pre-test and post-test (*0.050  >  p  ≥ 00.001; **p  < 0.001)

Number sign indicate a tendency for difference between pre-test and post-test (^#^0.100  >  p  ≥ 0.050)

#### Insulin-like growth factor 1

An overview of the IGF-1 values can be found in Table 3. For the IGF-1, a significant interaction effect (F_2,59_  = 5.964, p  = 0.005, η_p_^2^  = 0.162) and main time effect (F_1,59_  = 22.965, p  < 0.001, η_p_^2^  = 0.280) were observed. Follow-up tests revealed that the IGF-1 concentrations were significantly increased after a single OSE or CSE session (aOSE: F_1,23_  = 20.279, p  < 0.001, η_p_^2^  = 0.469 and aCSE: F_1,23_  = 20.217, p  < 0.001, η_p_^2^  = 0.468, respectively). In addition, the absolute changes in the IGF-1 were significantly higher in the aOSE and the aCSE group compared to the CG (see Fig. 4).

#### Interleukine-6

**Fig. 5:**
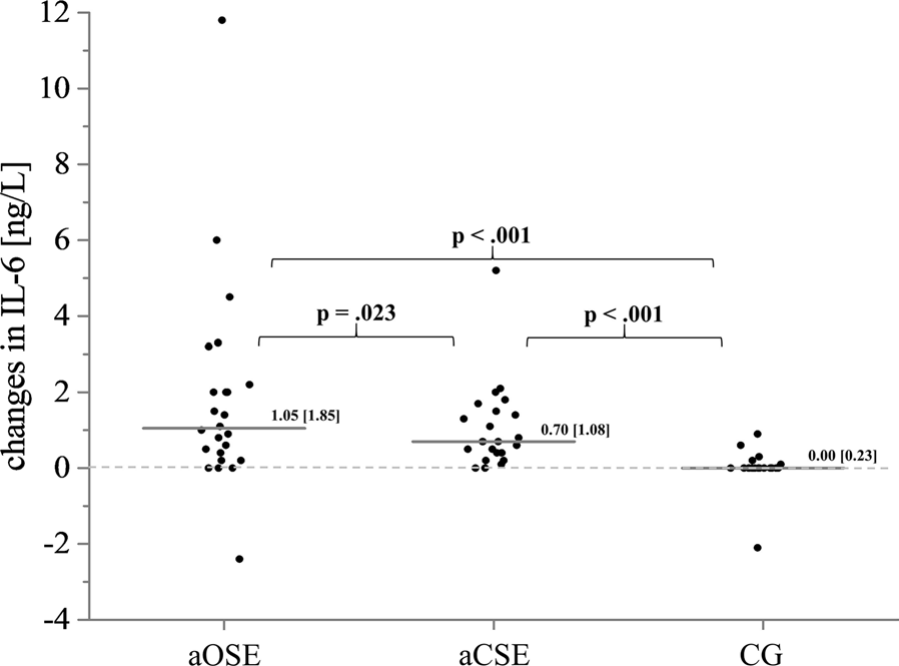
Median [grey line, median (interquartile range)] and intraindividual changes (black points) in serum IL-6 for the acute open-skill exercise group (aOSE: badminton), the acute closed-skill exercise group (aCSE: bicycling) and the control group (CG: reading)

Outcome values regarding IL-6 are shown in Table 4. Since no differences between groups were found during the pre-test, an initial homogeneity can be assumed. IL-6 values increased significantly from pre-test to post-test (time effect) in the aOSE and the aCSE group (Z  = − 3.460, p  = 0.001, r  = 0.706 and Z  = − 4.110, p  < 0.001, r  = 0.839, respectively). In post-test, participants from the aOSE and the aCSE group showed significantly higher IL-6 levels compared to the participants of the CG (Z  = − 3.230, p  =  0.001, r  = 0.524 and Z  = − 3.136, p  = 0.002, r  = 0.509, respectively). Statistical analyses of absolute changes in IL-6 concentration indicates a significant larger increase in the aOSE and the aCSE group compared to the CG. In addition, the absolute increase in IL-6 was significantly higher in the aOSE condition compared to the aCSE condition (see Fig. 5).

**Table 4:** Chronic effects of OSE and CSE on blood levels of BDNF_P_, BDNF_S_, IGF-1 and IL-6

Variable	cOSE			cCSE		
	Pre	Post	Change	Pre	Post	Change
BDNF_P_ (pg/ml)	1695.0 (837.5)	1810.0 (760.0)	+ 35.0 (1392.5)	1620.0 (810.0)	1850.0 (2168.0)	+ 63.0 (1739.0)
BDNF_S_ (pg/ml)	25,200.0 (4800.0)	27,400.0* (7650.0)	+ 2450.0 (4625.0)	30,700.0 (6450.0)	32,400.0 (14,050.0)	+ 3600.0 (11,500.0)
IGF-1 (ng/ml)	209.5 (96.8)	158.5* (90.3)	− 34.5 (34.25)	180.0 (81.0)	146.0^#^ (63.0)	− 21.0 (86.5)
IL-6 (ng/L)	1.6 (1.6)	5.4* (2.4)	+ 3.8 (2.8)	1.5 (4.7)	5.9 (1.4)	+ 4.0 (5.4)

Median values (interquartile range) of BDNF_P_, BDNF_S_, serum IGF-1, and serum IL-6 at pre-test and post-test

*BDNF* brain-derived neurotrophic factor; *IGF-1* insulin-like growth factor 1; *IL-6* interleukine-6

Asterisk indicates a significant difference between pre-test and post-test (*0.050 >  p  ≥ 0.001)

Number sign indicates a tendency for difference between pre-test and post-test (^#^0.100 >  p  ≥ 0.050)

### Chronic effects

**Fig. 6:**
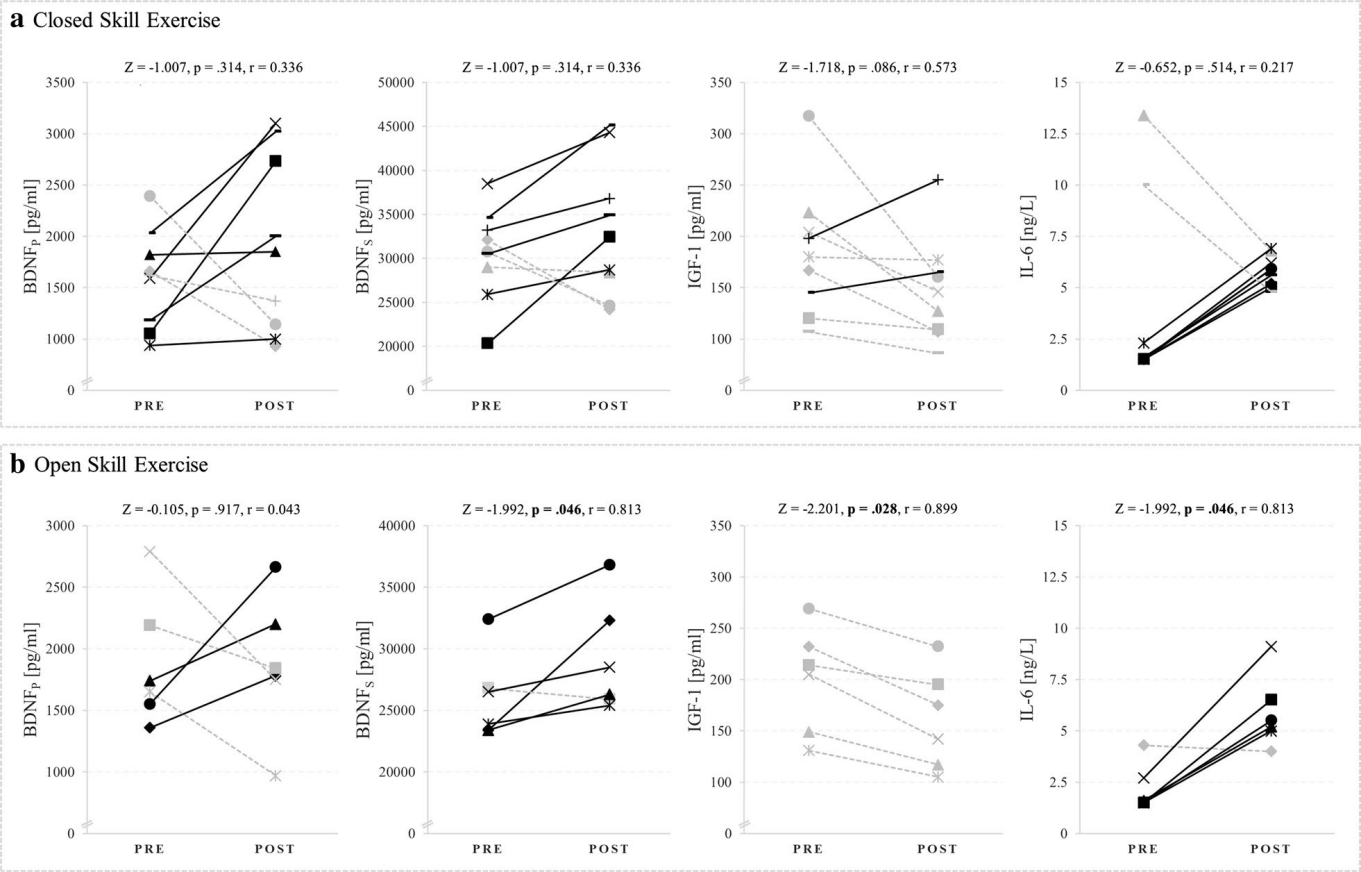
Absolute values of BDNF_P_, BDNF_S_, serum IGF-1 and serum IL-6 from pre-test (T1: before intervention) to post-test (T3: after 12 weeks of intervention) for the **a** cOSE-group and the **b** cCSE-group. The symbols in **a** and **b** represent one and the same person

Seven participants were excluded from data analyses due to injuries or low attendance rate (under 75%). This left a total of 15 participants (9 in the cCSE group and 6 in the cOSE group). Due to the small remaining sample size, we plotted the pre- and post-training levels of BDNF_P_, BDNF_S_, IGF-1 and IL-6 in Fig. 6 to better illustrate the results. The average attendance rate of the remaining participants from the oCSE and cCSE group was 84.7 ± 7.5% and 91.7 ± 7.9%, respectively. Except for body height, the participants in both groups exhibited comparable characteristics (see Table 2).

#### Brain-derived neurotrophic factor

Regarding BDNF_P_ and BDNF_S_, we did not find any between group differences at pre-test (Z  = − 0.825, p  = 0.456 and Z  = − 1.651, p  = 0.607) or at post-test (Z  = − 0.236, p  = 0.864 and Z  = − 0.885, p  = 0.388) (see Table 4). In addition, no time effects were observed for BDNF_P_ (cOSE: Z  = − 0.105, p  = 0.917; cCSE: Z  = − 1.007, p  = 0.314). For BDNF_S_, while no time effect for the cCSE group was observed (Z  = − 1.007, p  = 0.314), a significant time effect with a large effect size was identified for the cOSE group, with higher BDNF_S_ levels at post-test compared to pre-test (Z  = − 1.992, p  = 0.046, r  = 0.813). The graphs in Fig. 6 show that changes in BDNF can be—depending on participant—in opposite directions. With the exception of BDNF_P_ in the cOSE group, an increase of basal BDNF levels can be seen for most of the participants. Not unexpectedly, the direction of change is for most participants identical for BDNF_S_ and BDNF_P_.

#### Insulin-like growth factor 1

IGF-1 showed no significant between group differences at pre-test (Z  = − 0.943, p  = 0.388) or at post-test (Z  = − 0.471, p  = 0.689) (see Table 4). Regarding the cOSE group, a significant decrease in the IGF-1 levels could be observed (Z  = − 2.201, p  = 0.028, r  = 0.899). The IGF-1 levels also decreased in the cCSE group. The decrease in the IGF-1 levels from pre-test to post-test failed to reveal a significant time effect (Z  = − 1.718, p  = 0.086). However, the statistical effect size was large (r  = 0.573). Figure 6 shows that basal IGF-1 levels decreased in all but two participants from the cCSE group, and was reduced in all participants of the cCSE group after 12 weeks of intervention.

#### Interleukin-6

No significant pre-test or post-test differences were observed for IL-6 between groups (pre-test: Z  = 0.000, p  = 1.000; post-test: Z  = − 0.730, p  = 0.689) (see Table 4). While there was no significant time effect for the cCSE group (Z  = − 0.652, p  = 0.514), a significant time effect was observed for the cOSE group, with higher IL-6 levels at post-test compared to pre-test (Z = -1.992, p = 0.046, r = 0.813). Figure 6 indicates 2 participants in the cOSE group with substantially elevated IL-6 levels at pre-test that seemed to be reduced back to normal levels after 12 weeks of intervention. When these two “outliers” with untypical high IL-6 levels are removed from the statistical analysis, the observed trend for the other participants towards an increase in IL-6 becomes statistically significant (Z  = − 2.371, p  = 0.018, r  = 0.896). For the cOSE group, a trend towards increased IL-6 post-test is obvious.

While such an analysis for different neurotrophic and immunomodulatory molecules in the blood of study participants might open up interesting perspectives for the overall evaluation of sport interventions our current sample size is too low to draw clear conclusions.

## Discussion

In this study, we compared the acute and chronic effects of OSE and CSE on plasma and serum levels of BDNF, serum IGF-1, and serum IL-6 in healthy older adults. These neurotrophic factors and cytokines are assumed to modulate specific molecular and cellular pathways promoting neuroplasticity, that can enable cognitive improvements and the maintenance of brain integrity [20, 23, 25, 44, 65–67]. Based on our results presented above, the main findings of our study were two-fold: first, we could observe that BDNF_P_, BDNF_S_, IGF-1, and IL-6 levels were increased in response to both exercise types (aOSE and aCSE) after a single session of 30 min. Differences between the exercise types could only be found for serum IL-6, suggesting that a single badminton session (aOSE) compared with a single bicycle session (aCSE) leads to an elevated IL-6 level in healthy older adults. Second, after 12 weeks of training, the basal levels of BDNF_S_ and IL-6 increased whereas the basal level of IGF-1 decreased in the cOSE group, without differences between groups.

### Acute effects

BDNF_S_ was significantly increased after both exercise types, suggesting that a single session of either an OSE (badminton) or a CSE (bicycling) could acutely increase peripheral BDNF_S_ levels in healthy older adults. Previous studies investigating the acute effect of a single exercise session also found elevated peripheral BDNF_S_ levels in older and younger healthy people [56, 68–73]. Hung et al. [56] compared the effect of a single badminton session with a single running session (30 min at 60% of HRR) on BDNF_S_ production in twenty young (23.15 years) healthy males and could observe similar effects. However, in this study, the authors reported that the badminton session resulted in a significantly higher BDNF_S_ level compared to the running session [56]. In our investigation, we could not observe any differences in BDNF_S_ levels between exercise types. Although, BDNF is also expressed in vascular endothelial cells [74] and the skeletal muscle cells [75], the most prominent source of BDNF is the brain, where it is expressed at highest levels in the hippocampus and the neocortex [76–78]. Since BDNF can cross the blood–brain barrier [79], Rasmussen et al. [80] estimated that in humans, at rest and during exercise, 70–80% of circulating BDNF in the blood stream (determined in venous blood) originates from the brain. Strong evidence suggests that the exercise-induced changes in circulating BDNF levels are affected by a host of factors including general exercise variables such as the type (e.g., cycling, playing badminton, dancing), the intensity (describes how strenuous the exercise is), and the duration (time period of a particular exercise or entire exercise session) as well as individual characteristics such as age [10, 23, 49, 73, 81–84]. Regarding the exercise variables, both qualitative characteristics (e.g., exercise type and complexity) and quantitative characteristics (e.g., intensity and duration) need to be considered to clarify the exercise dose [10, 85, 86]. According to the distinction between OSE and CSE, the classification is based on qualitative exercise variables such as the predictability of the performing environment (dynamic and less predictable vs. static and mostly predictable environments) and the exercise complexity (coordinative and/or cognitive demands) [50, 87]. To take into account the differences between OSE and CSE in terms of their beneficial effects on cognitive functions and the underlying molecular and cellular mechanisms, all relevant quantitative exercise characteristics must be set equally. In the study of Hung et al. [56] as well as in our study, exercise duration (30 min) and intensity (average heart rate was used as a proxy for the internal load) were matched between OSE and CSE. However, while moderate continuous running and/or bicycling (CSE) are predominantly repetitive (cyclic) and internally-paced movements, badminton (OSE) is more externally-paced and consists of dynamic movements that, among other things, manifest themselves through rapid changes in movement direction and speed [88–90]. Therefore, it could be assumed that the internal load (as a proxy of exercise intensity) remains relatively constant during running or bicycling, while it fluctuates during a badminton session. Regarding the findings of Hung et al. [56], peaks in internal load during the badminton session could be one reason why BDNF_S_ levels were significantly higher compared to running. Compared to Hung et al. [56], our participants were much older and probably less agile. Thus, in our older cohort there were less pronounced fluctuations in internal load during the badminton session which could be one possible reason, among other factors, why we did not observe statistically significant differences in BDNF_S_ levels between the aOSE and the aCSE group. However, this remains speculative.

It should be noted that we did not screen our participants for symptoms of depression and therefore we cannot rule out an influence on our results due to underlying depressive disorders. Previous studies have shown decreased basal levels of BDNF_S_ in elderly people with depression compared to people without depression [91, 92]. Indeed, decreased BDNF_S_ levels may contribute to atrophy of certain limbic structures, including the hippocampus and prefrontal cortex, which have been observed in depressed adults [93, 94]. However, the mean values and standard deviations of the basal BDNF_S_ and BDNF_P_ levels presented in our study are comparable to the results of existing studies with healthy elderly adults [45, 95, 96].

While levels of BDNF_S_ reflect BDNF that is stored in platelets, BDNF_P_ concentrations reads out the freely circulating BDNF in the absence of platelet activation [34]. We observed that BDNF_P_ levels increased after both acute exercise session (aOSE: significant time effect; aCSE: tendentious time effect). However, in contrast to BDNF_S_, there were no differences, neither between the active groups (aOSE vs. aCSE) nor between any active group and the inactive CG (aOSE vs. CG and aCSE vs. CG). Recent studies have shown that BDNF_P_ levels did not significantly increase after a single session of aerobic [97–99] or resistance exercises [100] in young and older participants. Nofuij et al. [98] observed a non-significant increase in BDNF_P_ immediately after, 30 min after, and 60 min after low (40% VO_2max_), moderate (60% VO_2max_), and high (100% VO_2max_) intensity exercise tests (performed on a bicycle), while BDNF_S_ was significantly increased immediately after the low intensity exercise test. However, Cho et al. [101] have shown that both, BDNF_S_ and BDNF_P_ are significantly increased immediately after a maximal treadmill test in 18 healthy male students. Furthermore, Rasmussen et al. [80] reported that BDNF_P_ is increased after 4 hours of ergometer rowing corresponding to an intensity 10–15% below the lactate threshold but not after 2 hours. It is conceivable that an high intra- and inter-individual variability, caused by the dynamics of BDNF uptake and release, or other variables, like training status or neurological status, might be among the factors that account for the above mentioned inconsistent study results regarding the plasma BDNF levels [99]. We can only speculate, that acute changes in BDNF_P_ between the active groups and the inactive control group could become statistically significant with a higher sample size or another exercise prescription (e.g., prolonged exercise duration).

With regard to IGF-1, we observed that a single badminton session (aOSE) as well as a single session of bicycling (aCSE) leads to an increased IGF-1 concentration in blood serum. Existing studies indicate that the exercise-related release of IGF-1 is highly variable and is a function of exercise intensity, duration and modality. While IGF-1 serum levels were increased after short bouts (10–22 min) of high- [28, 70, 102] and low-intensity [28] cycling, other studies have reported a non-significant increase in serum IGF-1 following a single session of moderate swimming [103] and high intensity bicycling [69]. However, there is also evidence that the acute exercise-related release of IGF-1 is transient, peaking after 5–10 min of exercise and return to baseline or to significantly lower levels within 30–60 min after exercising [28, 104, 105]. Berg and Bang [104] suggested that the rapid increase in serum IGF-1 concentration following acute exercise is due to the release of IGF-1 from tissue stores (i.e., in the skeletal muscle) rather than to the peptide synthesis in the liver or muscle cells [67]. While findings of previous studies indicate that increased basal IGF-1 levels after long-term (24 up to 52 weeks) resistance training improve behavioural (several cognitive functions) and physiological (P3-amplitude) indicators for brain function [106, 107], an acute increase in IGF-1 following a single exercise session does not correlate with functional improvements [105]. These results suggest that the beneficial effect of IGF-1 relies on long-term mechanisms such as angiogenesis, and potentially, the regulation of synaptic plasticity in the brain [44, 67, 108–110].

Regarding IL-6, our results show an acute exercise-related increase in IL-6 in both active groups, with significantly higher changes in the aOSE group. Strong evidence suggests that IL-6 is expressed by myocytes in contracting muscle fibres e.g., due to physical exercising [111–113]. Interestingly, IL-6 is also expressed by the brain during prolonged physical exercise [47, 114], for instance, as a signal of metabolic stress within the brain or as a consequence of increased brain activity [33, 115]. Chronically elevated levels of IL-6 are often associated with adverse health effects (e.g., cognitive declines, metabolic disorders). Importantly, results from human and animal studies showed that IL-6 stimulates the production of anti-inflammatory cytokines (e.g., IL-10) [33, 48], supresses expression of tumor necrosis factor-α [48], regulates neuronal functions [24], stimulates neurogenesis (i.e., the process of creating new neurons and glial cells from stem cells) [24] and acts neuroprotective [116]. Therefore, the acute exercise-induced release of IL-6 could be involved in mediating some positive effects for brain health and cognitive functions. It is well known that the exercise type, intensity, duration as well as the mass of recruited skeletal muscles determines the magnitude of the acute exercise-induced release of IL-6 [48, 115, 117]. Since bicycling involves a limited skeletal muscle mass (lower extremities) and badminton involves nearly all muscle groups of the whole body, it is not surprising that the aOSE group leads to a significantly higher release of IL-6 into the blood compared to the aCSE group. Hence, we speculate that the group differences for acute changes of IL-6 (aOSE: badminton vs. aCSE: cycling) could be explained by the magnitude of involved musculature rather than by the inherent differences between OSE and CSE (i.e., predictability of the performing environment and the exercise complexity).

### Chronic effects

After 12 weeks of training, basal BDNF_S_ levels were increased in both groups (cOSE: + 2450.0 pg/ml; cCSE: + 3600.0 pg/ml). Although the absolute changes in the cCSE group are greater than in the cOSE group, only the peripheral BDNF_S_ levels of the cOSE group changed significantly from pre-test to post-test. The majority of previous studies investigating the chronic effects of regular physical-exercise (i.e. training) on basal levels of peripheral BDNF concentrations focused on aerobic and strength training with inhomogeneous results [49, 51, 81, 82, 118, 119]. So far, existing studies report unchanged [45, 120, 121], increased [83, 122, 123] and reduced [71, 124, 125] basal BDNF_S_ and BDNF_P_ levels in older individuals following an aerobic and/or resistance training. Additionally, a current meta-analysis by Marinus et al. [49] indicates that a combined aerobic-strength training is more effective in increasing peripheral BDNF concentrations in the elderly population than an isolated aerobic training. However, there is no consensus about the most beneficial exercise variables (intensity, duration, type of training) and training variables (frequency, density, period) [10] to induce an increase in peripheral BDNF concentrations. Regarding the exercise type, Grégoire et al. [126] observed that after 8 weeks of participation, a one-hour training intervention performed three times a week and including gross motor activities (juggling and ball-throwing exercises towards a target) induced a greater increase in basal BDNF_P_ levels than interventions including aerobic and strength training units in older healthy people. In addition, Rehfeld et al. [127] noticed in their training intervention study (6 month, 2 ×  per week, 90 min) that dancing increased basal BDNF_P_ levels in older healthy adults more than a health-related exercise training (including aerobic, strength and flexibility training units). In the same study the increase in grey and white matter volume in multiple brain areas was more pronounced in the dancing group (see also: [95, 128]). These results support the hypothesis, that physical exercises with sensory enrichment and coordinative-cognitive demands (e.g., OSE like dancing or badminton) have more pronounced effects, compared to physical exercises with no or low sensory and coordinative demands, respectively (e.g., CSE like an combined strength-endurance training), on neurophysiological mechanisms (e.g., the release of neurotrophic factors such as BDNF) and structural brain changes [95, 129]. In our study, we observed unchanged BDNF_P_ levels and increased BDNF_S_ levels (only statistically relevant for the cOSE group). However, we could not find any differences between the groups, neither for absolute values at post-test nor for the absolute changes from pre-test to post-test. This lack off an effect might result from two factors: (i) the low number of study participants and/or (ii) an insufficient “dose” of the physical exercise/training regime which depends on exercise prescription (e.g., exercise duration, training frequency, and training density) [10].

Although physical training, especially strength training, is traditionally associated with increased concentration of growth factors such as IGF-1 in the blood stream, we could not observe an increase in basal IGF-1 concentrations in blood serum for both groups. To the contrary, we observed in both groups a decrease of basal IGF-1 levels at the end of the 12-week intervention (with statistically significant changes in the cOSE group and a tendency towards significance in the cCSE group). This finding is in accordance with the observation of comparable studies which reported a decrease of basal IGF-1 levels after endurance training and strength training for young individuals [130] and older individuals [131]. In this regard, it should be noted that the rate of synthesis of IGF-1 does also depend on non-exercise related factors, such as the nutritional status [104], age [132] and availability of human growth hormone (GH) [133]. In general, a positive neurocognitive effect of IGF-1 has been observed in several intervention studies, in which a training-induced (aerobic and strength training) increase in basal IGF-1 concentration was associated with improved cognitive functions in older individuals [106, 134, 135]. In particular, in older individuals, several studies reported that an increase in basal levels of IGF-1 in response to a long-term aerobic training intervention is associated with improved functional connectivity, cognitive functions, and increased hippocampal volume, although the basal concentration of this growth factor was not affected by the training intervention [45, 131]. One might speculate that repeated rapid short-term changes in neuropeptides such as IGF-1 during and following physical exercises can trigger neurobiological processes that can converge into various neurocognitive changes (e.g., angiogenesis, structural and functional brain changes) if those short-term changes occur over a distinct time period repetitively. Since the exact mechanisms that explain the relationship between the exercise-induced alterations of IGF-1 and neurocognitive processes are sparsely understood [25, 32], this is an unresolved issue. With regard to the comparison between the cOSE and cCSE group, our study results do not show any advantages of either training or exercise type.

With regard to IL-6, our results indicate that the basal IL-6 concentration in blood serum increased in both groups (cOSE: + 3.8 ng/l; cCSE: + 4.0 ng/l), whereas only changes from the cOSE group became statistically significant. The possible lack of significant changes in IL-6 for the cCSE group might be due to the relatively small sample size and relatively high inter-individual differences at pre-test (see interquartile range and Fig. 6). Contrary to our results, Forti et al. [121] showed that basal IL-6 levels significantly decreased after 12 weeks of strength training (70–80% 1-RM, 3 ×  per week) in 20 older adults. However, the reported absolute changes were relatively small, with a median value of approximately 0.45 ng/l (absolute changes) and the authors could not observe significant differences in changes of IL-6 in comparison to an inactive control group. Other studies, performing a strength training with elderly participants, did not detect statistically significant changes in basal IL-6 levels after six (3 × 10 repetitions of 50% up to 70–80% 1-RM, 2 ×  per week [136]) and ten (3 ×  8 repetitions of 70–80% 1-RM, 2 ×  per week [137]) weeks of training. While prolonged and exacerbated IL-6 exposure is associated with inflammatory processes and numerous chronical disease such as dementia [138], it is not clear whether IL-6 is the cause or only a marker of disease [47, 139]. During physical exercise, peripheral IL-6 concentration was reported to increase acutely and returning to baseline level within 24 h [117, 140]. In this context, IL-6 is rather assumed to have an anti-inflammatory effect and could be a factor by which regular exercise might reduce chronic (low-grade) inflammation and the risk for neurological disorders [115, 116, 141]. Since IL-6 plays multiple roles in a wide array of biological processes in the human organism [33, 48, 142, 143], further research is needed to gain a better understanding of the relationship between exercise/training, the acute/chronic alterations of IL-6 and its effect on neurocognitive processes.

However, the present study is not without limitations that we have to acknowledge. First, we admit that there could be a possible bias due to the fact that badminton (aOSE) involves whole-body movements with weight-bearing whereas bicycling (aCSE) involves mainly lower-body movements without weight-bearing. Therefore, it might be possible that the muscle mass, which is involved in the body movement, may affect the acute exercise-released response of physiological markers (e.g., BDNF, IGF-1, IL-6). On the one hand, we chose badminton as OSE because it represents an economic and popular sports game, which involves physical as well as cognitive demands [90, 144]. On the other hand, due to our older participants, we chose bicycling instead of treadmill running to reduce the risk of falls and injuries. Secondly, although we have checked the average heart rate, subjective perception of effort, and blood lactate concentration during or immediately after each exercise, to ensure equal intensities between the aOSE and the aCSE group, we cannot be sure that during the exercise session, the internal load was exactly the same. This limitation should be taken into account when designing future studies. Third, the exercise sessions from the first investigation (acute effects; aOSE: badminton and aCSE: bicycling) and the second investigation (chronic effects; cOSE: performing different sport-games and cCSE: combined strength-endurance training) are not completely comparable without limitations. During the acute exercise sessions, intensity of exercise was set to 60 ± 5% of participants individual HRR and the heart rate was continuously controlled using a heart rate monitor. In the second investigation, the training intensity was individually adjusted to the subjective perceived exertion of the participants using the CR-10 Scale. Training intensity was individually adjusted so that each participant rated the intensity as a 7 out of 10. We have chosen to use the CR-10 scale for the chronic intervention because of methodological reasons. According to the American College of Sports Medicine position stand [145] and Noble et al. [62], both descriptions of intensity (60 ± 5% of HRR and a score of 7 on the CR-10 scale) are defined as vigorous exercise intensity (i.e., hard to very hard). However, the first and second investigation (i.e., acute vs. chronical) differ in the type of exercise/training (e.g., endurance exercise vs. combined strength-endurance exercise/training). Therefore, conclusions with regard to acute to chronic effects or vice versa should be interpreted cautiously. Finally, a high number of dropouts during the intervention study left a relatively small number of participants which could be analyzed.

## Conclusion

In conclusion, the present study examined possible differences between acute and chronic effects of OSE and CSE on BDNF_S_, BDNF_P_ as well as IGF-1 and IL-6 serum levels. We observed that both exercise types were equally efficient to acutely increase the BDNF_S_, BDNF_P_, IGF-1 and IL-6 serum levles in healthy older adults. Compared with cCSE, cOSE led to a significant increase in basal BDNF_S_. Although our results tend to support the notion that OSE training is superior in improving some aspects of structural and/or functional brain integrity compared to CSE, it is important to interpret the results with caution due to the relatively small number of participants in the chronical intervention and individual responses, especially regarding the BDNF levels. To prove whether OSE is more effective to enhance or maintain brain health than CSE, further studies are needed that, (i) control for qualitative and quantitative exercise and/or training variables, (ii) measure acute and/or chronic changes of neurotrophic factors with cellular resulution, (iii) determine structural and functional parameters (e.g., hemodynamic response, brain morphology), (iv) analyze behavioural, socioemotional (e.g., sleep, mood), and cognitive functions (e.g., executive functions, memory [20]). Moreover, evaluating of further cohorts (e.g., people with mild cognitive impairments, experts in badminton) is needed to obtain a more general picture regarding possible benefits of OSE over CSE.

## Data Availability

The datasets generated and/or analyzed during the current are kept in the Department of Sport Science, Otto von Guericke University Magdeburg and are available from the corresponding author on reasonable request.

## Abbreviations

ANOVA: Analysis of variance
BDNF: Brain-derived neurotrophic factor
BDNFs: Serum Brain-derived neurotrophic factor
BDNF_P_: Plasma Brain-derived neurotrophic factor
CR-10: Category Ratio-10 Scale
CSE: Closed-skill exercise
HR_max_: Maximum heart rate
HRR: Heart rate reserve
HR_rest_: Resting heart rate
IGF-1: Insulin-like growth factor 1
IL-6: Interleukin-6
MMSE: Mini-mental State Examination
OSE: Open-skill exercise
RPE: Rating of perceived exertion
η_p_^2^: Partial eta-squared
